# Therapeutic Note

**Published:** 1906-08

**Authors:** 


### Therapeutic Note.

### THE PROPER STRENGTH OF ADRENALIN SOLUTIONS IN THE TREATMENT OF HAYFEVER.

In the treatment of hay fever with adrenalin chloride, it has been suggested that weak solutions, frequently applied, are apt to yield better results than the occasional application of a strong solution. One of the pathological features of this peculiar malady is a turgescence of the turbinal tissues due to excessive dilation of the capillaries. That this is the result of a neurosis involving more or less pronounced vasomotor paralysis is pretty generally conceded. Overstimulation, by reaction, is very sure to result in a complete paralysis of the vasomotor supply in the region affected. On the other hand, gentle stimulation with weak solutions is not so likely to be followed by reaction.

These views are in harmony with the published observations of Crile, of Cleveland, who found that in a decapitated animal the heart's action was better sustained by the continuous administration of a weak solution of adrenalin chloride. Furthermore, this is probably nature's method of supplying this vital principle to the healthy human body through the agency of the suprarenal gland, its constant presence in the blood in minute amounts being sufficient to maintain vasomotor equilibrium.

